# Incidence of Symptomatic Venous Thromboembolism (VTE) in 8,885 Elective Total Hip Arthroplasty Patients Receiving Post-operative Aspirin VTE Prophylaxis

**DOI:** 10.7759/cureus.36464

**Published:** 2023-03-21

**Authors:** Eamonn I Coveney, Christopher Hutton, Nimesh Patel, Sarah L Whitehouse, Jonathan R Howell, Matthew J Wilson, Matthew J Hubble, John Charity, Al-Amin M Kassam

**Affiliations:** 1 Exeter Hip Unit, Royal Devon University Healthcare NHS Foundation Trust, Exeter, GBR; 2 Orthopaedic Research Unit, Queensland University of Technology, Brisbane, AUS

**Keywords:** vte prophylaxis, total hip athroplasty, aspirin, venous thromboembolsim, cemented total hip arthroplasty

## Abstract

Background: Venous thromboembolism (VTE) is a potentially reducible cause of morbidity and mortality in patients undergoing elective hip arthroplasty surgery. The balance of post-operative VTE prophylaxis and risk of post-operative haemorrhage remains at the forefront of surgeon’s mind. The National Institute for Health and Care Excellence (NICE) published updated guidelines in 2018 which recommend the use of both mechanical and pharmacological methods in patients undergoing elective total hip arthroplasty (THA).

Objectives: The aim of this study was to present the symptomatic VTE incidence in 8,885 patients who underwent THA between January 1998 and March 2018 with Aspirin as the primary agent for pharmacological thromboprophylaxis. Intermittent calf compression stockings are routinely used from the time of surgery until mobilization (usually the following day) with prophylactic doses of low molecular weight heparin (LMWH) during inpatient stay (from 2005 onwards) and then Aspirin 150mg once daily for six weeks on hospital discharge (or Aspirin only prior to 2005), with use of other therapies occasionally as required.

Methods: Analysis of prospective data collection from consecutive patients at a single institution undergoing THA was performed with the incidence of symptomatic deep vein thrombosis (DVT) and pulmonary embolism (PE) occurring within six months of the index operation as the primary outcome measure. Ninety-day all-cause mortality of this cohort of patients was also analysed.

Results: 8,885 patients were reviewed. This included 7230 primary, 224 complex primary and 1431 revision cases. The overall incidence of symptomatic VTE after elective THA was 1.11% (99/8885) - with the incidence of symptomatic DVT of 0.59% (52/8885) and the incidence of symptomatic PE of 0.53% (47/8885). There was no significant difference (χ^2^ test, p=0.239) in the symptomatic VTE incidence between primary (1.20% - 89/7230), complex primary (0.89% - 2/224) and revision cases (0.70% - 10/1431). The 90-day all-cause mortality was 0.88% (78/8885). Cardiovascular and respiratory disease were the main causes of death following surgery. Only 0.03% of deaths (n= 3) within 90 days of index surgery were due to PE. There was no significant difference (p=0.327) in length of stay (and hence amount of pharmacologic prophylaxis with LMWH received by patients before commencement of Aspirin) with the average length of stay for those patients who did not suffer a VTE of 6.8 days compared with 7.6 days for those who did suffer a VTE.

Conclusion: Our results support the use of aspirin as an effective form of prophylaxis against symptomatic VTE following THA in contradiction to NICE and American Academy of Orthopaedic Surgery (AAOS) recommendations. It is not associated with an increased incidence in symptomatic DVT, PE or death compared to other published studies. The fact that it is inexpensive, readily available, requires no monitoring and does not pose an increased risk of bleeding are other advantages of using aspirin for VTE prophylaxis.

## Introduction

Venous thromboembolism (VTE) is a potentially preventable cause of morbidity and mortality in patients undergoing elective total hip arthroplasty (THA) surgery. In the absence of prophylaxis, it has been documented that VTE may occur in greater than 35% of patients undergoing arthroplasty surgery [1]. In the presence of VTE after hip or knee arthroplasty, mortality can be as high as 7% [2]. Choosing a venous thromboprophylaxis regime is therefore a very important and relevant topic amongst orthopaedic surgeons.

The balance of post-operative VTE prophylaxis and risk of post-operative haemorrhage remains at the forefront of the surgeon’s mind. In addition to pharmacological agents, a combination of mechanical prophylaxis, early mobilisation and modern surgical techniques are utilised to reduce the risk of VTE [3].

In 2007 the National Institute for Health and Care Excellence (NICE) published guideline CG46 which recommended offering patients undergoing elective orthopaedic surgery mechanical prophylaxis and chemoprophylaxis with either fondaparinux or low molecular weight heparin (LMWH) for 28 days post-operatively [4].

Further to this, the American Academy of Orthopaedic Surgeons (AAOS) created a working group in 2011 on VTE. They also suggested the use of mechanical and/or pharmacologic agents for the prevention of VTE in patients undergoing elective hip or knee arthroplasty. However, they stated that current evidence was unclear about which prophylactic strategy is optimal for the prevention of VTE [3].

NICE published updated guidelines in 2018 that recommend the use of both mechanical and pharmacological methods in patients undergoing elective hip arthroplasty surgery. They advocate either LMWH for 10 days followed by Aspirin for 28 days, LMWH for 28 days or a Factor Xa inhibitor (rivaroxaban) for 28 days [5].

There are multiple studies that document the effectiveness of Aspirin in reducing the incidence of VTE following elective arthroplasty surgery [6-10]. The popularity of Aspirin as a pharmacological agent for VTE prophylaxis, amongst Orthopaedic surgeons, was demonstrated at the 2016 American Association of Hip and Knee Surgeons annual meeting where more than 80% of 1200 delegates revealed that they use Aspirin as the primary agent [11]. This was further strengthened by a recent International Consensus Meeting which reached strong consensus on prescribing low-dose Aspirin as prophylaxis in hip and knee arthroplasty [12]. Further benefits of Aspirin include its low cost, the fact that it is well tolerated and does not require regular monitoring. Despite this clinical consensus from specialists in the field, the scientific evidence behind this consensus is lacking and there is still a reluctance in some units to employ Aspirin as sole VTE prophylaxis due to concerns about its efficacy compared to novel oral anticoagulants or warfarin.

The aim of this study is to investigate the incidence of symptomatic VTE in patients undergoing primary and revision total hip arthroplasty in our tertiary hip unit where Aspirin has been used as the primary agent for pharmacological thromboprophylaxis since 1997.

## Materials and methods

We performed an analysis, after institutional ethical board approval, of prospectively collected data, on patients undergoing primary THA, including complex primary THA, and revision THA between January 1998 and March 2018. Data including patient complication questionnaires, including specific questions about VTE, bleeding events and wound discharge, is routinely collected postoperatively. Other data collected included patient demographics, surgical intervention, morbidity and mortality. All primary THA cases were included. Patients with an incomplete patient-completed complications questionnaire were excluded rather than assuming no VTE had occurred. Sensitivity analysis was performed to ensure these excluded cases were similar to the included cohort.

Positive cases were defined as symptomatic VTE within a six-month period of the index procedure. This was broken down into incidence of deep vein thrombosis (DVT) and pulmonary embolus (PE).

**Figure 1:**
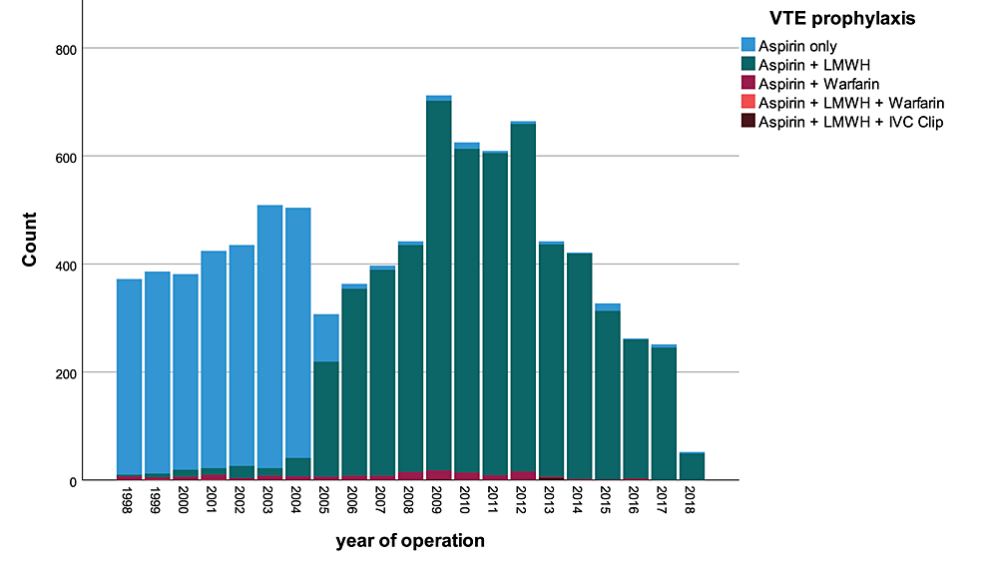
Stacked histogram of VTE prophylaxis use over time VTE, venous thromboembolism; LMWH, low molecular weight heparin; IVC, inferior vena cava

Following recommendations from the Scottish Intercollegiate Guidelines Network (SIGN) and PEP study [7], our standard VTE prophylaxis protocol from January 1998 was to use intermittent calf compression stockings from the time of surgery until mobilisation (usually the following day) unless contraindicated (Table 1, Figure 1) with Aspirin 150mg once daily for six weeks. This was updated in 2005 with the addition of prophylactic doses of LMWH administered during inpatient stay which were then switched to Aspirin 150mg once daily for six weeks on hospital discharge. Patients with a previous history of proximal DVT or PE, or those who were assessed as high risk (e.g. bilateral simultaneous THA or active bowel cancer) were discharged with warfarin instead of Aspirin (LMWH was used initially until the international normalized ratio (INR) reached therapeutic range). The VTE prophylaxis protocol has always contained Aspirin in our unit since 1997 despite NICE and AAOS guidelines.

**Table 1:** Frequency of therapies used LMWH, low molecular weight heparin; IVC, inferior vena cava

Therapy used	Frequency %)
Aspirin only	3035 (34.2%)
Aspirin + LMWH as inpatient	5697 (64.1%)
Aspirin + Warfarin	146 (1.6%)
Aspirin + LMWH + Warfarin	2 (0.02%)
Aspirin + LMWH + IVC Clip	5 (0.06%)
Total	8885

All surgeons undertaking THA were included in the analysis. The procedure was undertaken using a standard posterior approach or piriformis sparing modification of the posterior approach. Patients were encouraged to mobilise on the day of surgery or day one post-operatively unless there were any contraindications.

Diagnosis of DVT was confirmed on duplex ultrasound scanning and PE was confirmed on ventilation perfusion (VQ) scanning or computed tomography pulmonary angiography (CTPA).

## Results

Of 10,795 patients who underwent elective THA between 1998 and 2018, 1,910 patients were excluded as the patient-completed complications questionnaire was incomplete, and therefore we could not accurately determine whether they had suffered a post-operative VTE. This was deemed preferable to assuming that no VTE had occurred which would potentially positively skew the results. Sensitivity analysis on this excluded cohort is presented later. This left complete data from 8,885 cases available for analysis.

**Figure 2:**
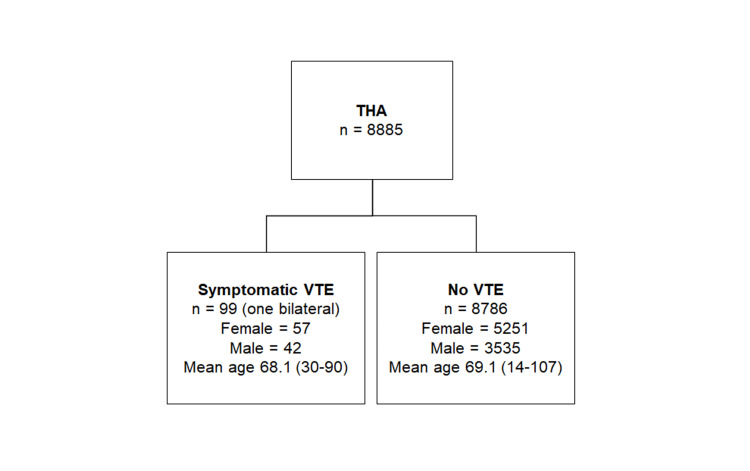
Flow chart showing the patient demographics THA, total hip arthroplasty; VTE, venous thromboembolism

Of the 8,885 patients reviewed, 7,230 had a routine primary THA (of which 262 had had previous, non-arthroplasty surgery), 224 were deemed complex primary cases and 1,431 were revision cases, including 15 Girdlestone procedures (Table 2). The incidence of symptomatic VTE was 1.11% (99/8885) after elective hip arthroplasty, with the incidence of symptomatic DVT 0.59% (52/8885) and PE 0.53% (47/8885). Of those patients who suffered a VTE, 57 were female and 42 were male with a ratio F:M 1.36:1 (Table 3, Figure 2; (χ2 test, p=0.662)). There was no significant difference (χ2 test, p=0.239) in the symptomatic VTE incidence between primary (1.20% - 87/7230), complex primary (0.89% - 2/224) and revision cases (0.70% - 10/1431) (Table 2).

**Table 2:** Surgery performed and incidence of symptomatic VTE THA, total hip arthroplasty; VTE, venous thromboembolism

Operation	N	Symptomatic VTE
Routine primary THA	7230	87 (1.20%)
Complex Primary THA	224	2 (0.89%)
Revision THA	1431	10 (0.70%)
Total	8885	99 (1.11%)

**Table 3:** Numbers of symptomatic DVT and PE by sex DVT, deep vein thrombosis; PE, pulmonary embolism

	Female	Male	Total
DVT	33 (0.62%)	19 (0.53%)	52
PE	24 (0.45%)	23 (0.64%)	47
Total	5308	3577	8885

The 90-day all-cause mortality was 0.88% (78/8885). Cardiovascular and respiratory disease were the main causes of death following surgery. Only 0.03% of deaths (n=3) within 90 days of index surgery were classified as due to PE. In the first 10-year period of data from 1998 to end of 2007 inclusive, the 90-day all-cause mortality rate was 1.13%. This fell significantly to 0.67% between 2008 and 2018 inclusive (Table 4; p=0.020 χ^2^test). Similarly, the rate of symptomatic VTE dropped significantly between these time periods (1.64% vs 0.67%; p<0.001 χ^2^test).

**Table 4:** Incidence of symptomatic VTE per year and 90-day mortality rate VTE, venous thromboembolism

Year	N	VTE	Deaths within 90 days	90-day mortality
1998	372	10 (2.69%)	2	0.54%
1999	386	11 (2.85%)	3	0.78%
2000	381	9 (2.36%)	5	1.31%
2001	424	7 (1.65%)	2	0.47%
2002	435	7 (1.61%)	9	2.07%
2003	509	5 (0.98%)	6	1.18%
2004	504	5 (0.99%)	6	1.19%
2005	307	7 (2.28%)	2	0.65%
2006	363	1 (0.28%)	4	1.10%
2007	397	5 (1.26%)	7	1.76%
2008	442	1 (0.23%)	3	0.68%
2009	712	9 (1.26%)	2	0.28%
2010	625	5 (0.80%)	3	0.48%
2011	609	6 (0.99%)	7	1.15%
2012	664	3 (0.45%)	3	0.45%
2013	442	0 (0.0%)	3	0.68%
2014	421	0 (0.0%)	3	0.71%
2015	327	1 (0.31%)	3	0.92%
2016	262	3 (1.15%)	4	1.53%
2017	251	3 (1.20%)	1	0.40%
2018 (to March)	52	1 (1.92%)	0	0.0%

The incidence of wound discharge longer than five days was 1.2% (105/8890) and for GI bleed was 0.6% (55/8890). There was one patient who suffered both a prolonged wound discharge and recorded a GI bleed.

The mean time for surgery was 105.3 minutes (SD 44.7, range 32-480) overall in the VTE group compared with 103.9 minutes (SD 36.6, range 66-300) in the no VTE group (p=0.697, t-test). There was no significant difference in surgery time for VTE compared with no VTE for routine primaries, complex primaries or revisions (Table 5).

**Table 5:** Mean (SD, range) time of surgery M-W U test is nonparametric Mann-Whitney U-test; THA, total hip arthroplasty; VTE, venous thromboembolism; SD, standard deviation

Surgery	Mean time in minutes (no VTE)	Mean time in minutes (VTE)	p-value
Routine primary THA	91.8 (21.7, 32-400)	96.4 (18.7, 66-175)	p=0.051 (t-test)
Complex primary THA	123.5 (41.4, 60-300)	80 (2 cases; 80, 200))	p=0.069 (M-W U test)
Revision THA	171.8 (66.7, 36-480)	181.1 (9 cases; 69.9, 90-300)	p=0.643 (M-W U test)

**Figure 3:**
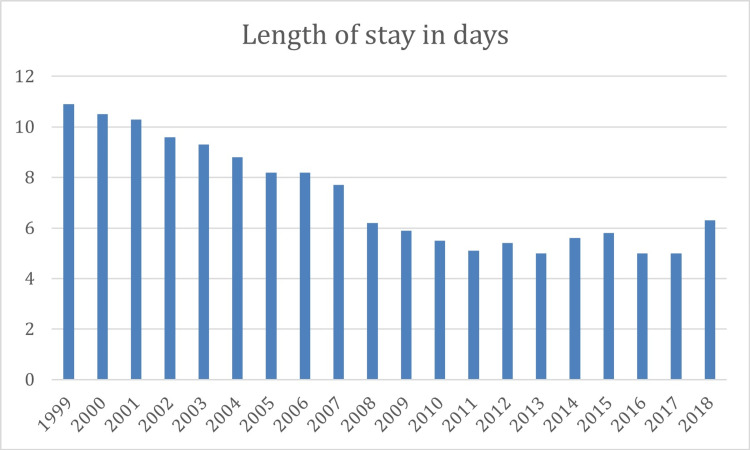
Length of stay over time

From the patient sample of 8,885 patients, length of stay data was available for 7343 patients from March 1999 to March 2018. This included 7318 patients who did not have a VTE and 25 patients who had a VTE. There was no significant difference in length of stay between those patients who did not suffer a VTE (6.8 days) and those that did (7.6 days; p=0.327) (Figure 3). Therefore, there was no difference in the amount of pharmacologic prophylaxis with LMWH received by patients before commencement of Aspirin.

In order to determine if the exclusion of cases without completed complication forms introduced any bias in the reporting, a sensitivity analysis was performed to examine 90-day mortality and reasons for death. Of the 1,910 cases, there were 14 deaths (0.73% - 14/1910) within 90 days which is comparable to the included cohort of 0.88% (78/8895; p=0.532 χ2 test). Two of these 14 deaths were attributable to PE, which was comparable to the rate for the included cases (0.10% - 2/1910 vs 0.03% - 3/8885; p=0.216 Fishers Exact test). 

## Discussion

Despite not adhering to the NICE and AAOS guidelines for chemical prophylaxis, our overall symptomatic VTE rates are comparable with international studies. Mula et al. report their incidence of VTE after THA of 1.2% [13].

Our use of Aspirin as the primary pharmacological prophylactic agent against VTE resulted in a 90-day mortality rate due to VTE of 0.03%. This is comparable to that of Cusick et al. who reported a rate of 0.05% in their cohort [14].

VTE prophylaxis in the post-operative period for patients has been a topical subject internationally, with various regimens being recommended in the setting of THA [3-5,15-17].

VTE has been highlighted as a potentially reducible cause of death and emphasised as a public health problem since the Parliamentary Health Committee Report in 2005 (https://publications.parliament.uk/pa/cm200405/cmselect/cmhealth/99/9902.htm). Following this NICE issued its initial guidelines to reduce VTE-related morbidity and mortality [4]. Since then, there has been widespread use of LMWH, warfarin and novel oral anticoagulants (NOACs) as part of a multimodal approach to reducing the incidence of VTE. With an update in NICE guidance in 2018, which did include off-label Aspirin, more surgeons have switched to using Aspirin in their VTE prophylaxis regimen in the setting of hip arthroplasty but after treatment with 10 days of LMWH [15]. In our unit, we only prescribe LMWH heparin whilst an inpatient with six weeks Aspirin prescribed on discharge.

Aspirin, when compared with other agents, has demonstrated lower mortality rates due to other causes rather than VTE when compared to other thromboprophylactic agents [18,19]. This reduction in mortality associated with Aspirin use is not unsurprising. The most common cause of mortality post arthroplasty is myocardial infarction. PEs are responsible for between 11.7% and 17.1% of deaths within 90 days post-arthroplasty compared to myocardial infarction being responsible for 25.9% of deaths within 90 days [20,21]. The antiplatelet effect of Aspirin has been shown to reduce the cardiac cause of death after THA [22].

Potent anti-coagulants are associated with a higher all-cause mortality rate after hip and knee arthroplasty, as well as increased risks of wound discharge, post-operative haematoma, lower limb oedema and potential for development of infection [10,19,20,23-26]. We are aware of well-documented side effects of gastrointestinal bleeds and with wound discharge post-operatively with Aspirin but it has a better side effect profile to other pharmacologic agents frequently used in VTE prophylaxis [24]. Our results regarding prolonged wound discharge in 1.2% are slightly superior (i.e. reduced) when compared with other studies. Singh et al. reported prolonged wound discharge rates of 3.2% [26]. Similarly, our gastrointestinal (GI) bleed rate of 0.5% is comparable with Gregg et al. who demonstrated a GI bleed rate of 0.6% in their cohort using Aspirin prophylaxis [27].

Prevention of VTE is not solely down to pharmacological measures. Increased awareness of mechanical prophylaxis methods, peri-operative patient nutrition and hydration optimisation, improved anaesthetic techniques and earlier mobilisation have all improved during the duration of our studied cohort. Our cohort reflects this as our all-cause mortality rate fell significantly between the periods of 1998-2007 and 2008-2017 from 1.13% to 0.67%. Our mean length of stay has reduced significantly (p<0.001) from 10.9 days in 1999 to 5.0 days in 2017 (latest full year in the cohort), which may seem high by current standards but are typical of the time periods included in this cohort. Despite patients receiving less in-hospital pharmacologic prophylaxis as a result of being discharged earlier, our VTE rates have also fallen from 1.71% in 1998-2007 to 0.83% in 2008-2017, again supporting the efficacy of Aspirin alongside mechanical measures as pharmacologic VTE prophylaxis.

When compared with warfarin, Aspirin is inexpensive and has been shown to be more cost-effective by reducing length of stay and lowering the risk of PE and other complications related to VTE prophylaxis [28]. This is even before the cost of monitoring with warfarin is considered both in hospital and in the community, which is time-consuming, requires invasive blood tests and is labour expensive. Jameson et al. (2014) have demonstrated that the annual cost of VTE prophylaxis using potent anticoagulants was approximately £13 million (using the example of Clexane) in the UK and Wales compared to the potential cost of £110,000 if aspirin had been used [29]. Aspirin has good compliance with patients as it is given as a once-daily oral dose, requires no monitoring and no need for the use of subcutaneous injection.

Many studies have shown that alternatives to Aspirin such as LMWH have increased risk of minor and major bleeding as well as wound complications [6,10]. Bloch et al. found that dabigatran caused a significant increase in leakage from the wound, an increase in the length of hospital stays and higher rates of VTE compared with the use of an LMWH inpatient and extended use of Aspirin [23]. Zou et al. (2014) found that rivaroxaban caused significantly greater hidden blood loss and more wound complications compared with LWMH and Aspirin when used as post-operative prophylaxis for total knee arthroplasty [24].

More recently, two systematic reviews of randomised control trials by Matharu et al. and Singjie et al. noted that Aspirin did not differ significantly from other anticoagulants in terms of safety and clinical effectiveness after THA [25,30]. However, the recent CRISTAL study from Australia reported significantly higher rates of symptomatic VTE with Aspirin (3.45%) compared with enoxaparin (1.82%), both of which were significantly higher than our reported 1.11%, with fewer patient numbers [16]. A study by Agarwal in 2023 in the US [17] reported decreasing trends of VTE and increasing use of Aspirin as a prophylactic agent.

The limitations of our study include that some data on our patients was incomplete resulting in exclusion (17.7%). The authors did not check deaths with the national death register which resulted in us having to trust the hospital system linked to General Practitioner databases. The authors also felt that exclusion of these cases was preferable to assuming that no VTE had occurred which would artificially lower the incidence of VTE. Although the characteristics of this cohort are not fully known, the 90-day mortality rate and reasons for death have been investigated and are comparable to the included cohort, giving some confidence in the decision to exclude. However, this is still one of the largest consecutive cohorts presented in the literature from a single unit with prospectively collected data and a sample size of 8885 patients across a 20-year period commencing before NICE guidelines were introduced.

## Conclusions

We have shown that Aspirin is an effective and safe method of symptomatic VTE prophylaxis following elective THA as a primary agent. We have demonstrated that its use is not associated with an increased incidence in symptomatic DVT, PE or mortality compared to other published studies, and it has comparable incidence of prolonged wound discharge and GI bleeds. We have also demonstrated that reducing our post-operative length of stay and, therefore, in-hospital pharmacological prophylaxis, has led to a reduction in VTE events. Our evidence supports the use of Aspirin as the main pharmacological prophylaxis in management of perioperative VTE risk in primary, complex primary and revision arthroplasty setting.
